# Decomposition Technique for Bio-Transmittance Imaging Based on Attenuation Coefficient Matrix Inverse

**DOI:** 10.3390/jimaging10010022

**Published:** 2024-01-15

**Authors:** Purnomo Sidi Priambodo, Toto Aminoto, Basari Basari

**Affiliations:** 1 Department of Electrical Engineering, Faculty of Engineering, Unversitas Indonesia, Kampus UI, Depok 16424, Indonesia; 2 Jakarta Polytechnic of Health III, Ministry of Health, Bekasi 405010, Indonesia

**Keywords:** biological tissue, transmittance image, image decomposition technique, monochromatic light, attenuation coefficient, matrix inverse, near-infrared

## Abstract

Human body tissue disease diagnosis will become more accurate if transmittance images, such as X-ray images, are separated according to each constituent tissue. This research proposes a new image decomposition technique based on the matrix inverse method for biological tissue images. The fundamental idea of this research is based on the fact that when *k* different monochromatic lights penetrate a biological tissue, they will experience different attenuation coefficients. Furthermore, the same happens when monochromatic light penetrates *k* different biological tissues, as they will also experience different attenuation coefficients. The various attenuation coefficients are arranged into a unique k×k-dimensional square matrix. k-many images taken by k-many different monochromatic lights are then merged into an image vector entity; further, a matrix inverse operation is performed on the merged image, producing *N*-many tissue thickness images of the constituent tissues. This research demonstrates that the proposed method effectively decomposes images of biological objects into separate images, each showing the thickness distributions of different constituent tissues. In the future, this proposed new technique is expected to contribute to supporting medical imaging analysis.

## 1. Introduction

Various cutting-edge tomography technologies are currently used in the medical field, including X-ray, computed tomography (CT) scan, single-photon emission computed tomography (SPECT), positron emission tomography (PET), and magnetic resonance imaging (MRI) [1,2,3,4,5,6]. These existing tomography technologies use high-energy media based on radioactive materials, magnetic fields, or high-frequency electromagnetic sources. Such high-energy media have a sizeable penetrating power and are able to render high-quality transmittance images. Transmittance images of human tissues provide more comprehensive information regarding the overall object’s volume than reflection images. The technique used to obtain a transmittance or a penetration image is called tomography [7].

The current sophisticated high-resolution medical imaging instruments are expensive and based on high-energy media, all with health risks. Developing a new tomography technique that uses a lower-energy medium and is less expensive is necessary. Several tomography methods have been proposed to meet these needs; some operate in the infrared region [8,9,10]. For consistency of the discussion and ease of understanding, the term tomography is replaced with transmittance image hereafter. Previous studies on infrared transmittance images have published various images of organ tissues in the body. A transmittance imaging apparatus generated a chicken bone image using a wavelength of 785 nm [11,12]. Images of hand veins, skin, and bones have been generated by taking transmittance images of the back of the hand using near-infrared (NIR) light at 850 nm [13].

This research proposes the use of a near-infrared (NIR) medium for its convenience, i.e., it is available for multiple wavelengths, nonradiative, and nonionizing. NIR also has the advantage of experiencing relatively low attenuation when penetrating biological tissues and a high attenuation ratio between different tissues. It provides 2D transmittance images with good contrast, wherein different tissues can be easily distinguished. Furthermore, to obtain clarity on a medical object, one study proposed a multimodality image fusion method using diversified modalities, such as CT, MRI, SPECT, PET, etc., combined and used for sharper image analysis, thereby increasing accuracy in clinical diagnosis [14]. However, to this day, the resulting 2D transmittance images have only been single transmittance images, displaying a superimposition of all constituent tissues of the captured object. So far, no attempt has been made by researchers to perform the separation of a captured image of a biological object into an image of each constituent tissue. The primary motivation for this research is to decompose a transmittance image to separate each constituent tissue. It is expected that in the future, decomposing bio-imaging into each constituent tissue image will aid doctors in diagnosing diseases that only affect one specific tissue. It is like diagnosis using MRI and CT scan results but using a low-energy medium, which is much safer.

This new technique for bio-transmittance-image decomposition is based on the fact that an NIR wavelength experiences different attenuation coefficients when penetrating other bio-tissues, and the illumination of different NIR wavelengths on the same bio-tissue results in different attenuation coefficients. This attenuation coefficient ratio is referred to as the contrast ratio [15]. The relationship functions of attenuation coefficients versus NIR wavelength and attenuation coefficients versus materials are then formed as an attenuation coefficient square matrix. The matrix inverse plays a vital role in the image decomposition process in this discussion.

Several researchers have previously developed methods for measuring the optical properties of biological tissues. The study cited in [16] reviews comprehensive optical properties such as absorption, scattering, total attenuation, effective attenuation, and anisotropy coefficients. Light propagation models are examined from the perspective of the Beer–Lambert law, diffusion theory, transport properties, and Kubelka–Munk coefficients. The method developed to measure the light attenuation properties of biological tissue is very important and should also be carried out in the long-infrared wavelength area. Brain tissue is one of the tissues that is an object of research and measurement of the attenuation coefficient in the spectrum range of 600–2400 nm [17]. However, the results of measuring optical coefficients on biological tissue often vary and are very dependent on the specimen’s humidity conditions. Furthermore, Ref. [18] proposed a method based on neural networks to help obtain more accurate optical coefficients of the tissues.

In illustrating how the transmittance image decomposition technique works, a biological object composed of k different tissues will be photographed as many as k times with *k* different NIR monochromatic wavelength illuminations in the same transmittance image frame. In this research, k is three, where the three tissues are chicken meat, skin, and bone merged into one single bio-object. When the three transmittance images are merged into one frame, each pixel point of the frame will consist of three different intensity values arranged as an image intensity vector. Each value of the image intensity vector is the result of a linear equation formed by the sum of the thickness of each tissue multiplied by the attenuation coefficient of its corresponding wavelength. If normalized to the light source, the image intensity vector becomes the attenuation vector. Hence, an image thickness vector in which the components represent the thickness of the tissue at a certain point can be obtained by operating the inverse attenuation matrix on the image attenuation vector. An image of tissue thickness can be formed in 2D from the thickness vector components associated with the tissue. The main innovation of this research is that we separate each image of tissue thickness by decomposing the original image taken by the camera. This innovation can be further developed for 3D image analysis of the biological objects under observation.

The experiments demonstrated successful results of image separation in the form of the thickness images of each constituent tissue. Although the results are still in progress, the developed technique works successfully. There is still much room for improvement in the future, especially in terms of increasing the accuracy and the contrast ratio between tissue images, for instance, by using machine learning methods [18]. Innovation in the development of this new technique of image decomposition is expected to aid doctors in diagnosing various problems in human body tissues.

## 2. Theoretical Development and Methods

### 2.1. Theoretical Development

Before explaining the image decomposition process with a matrix inverse, it is necessary to explain the attenuation process of electromagnetic wave penetration in a medium. Electromagnetic waves experience attenuation while penetrating a medium. Attenuation is the result of absorption and scattering in the medium. The amount of attenuation is determined by the medium’s material type, penetrating wavelength, and thickness *d*. In general, the attenuation relationship can be expressed by the Beer–Lambert law [19,20]:

(1) $$I=I_{0}e^{-{µ}_{\lambda }d}$$

where *I*_0_ is the intensity of the light as it enters the medium; *I* is the intensity of light after passing through a medium with thickness *d*; and $\mu_{\lambda }$ is the medium attenuation coefficient, which is a function of the optical wavelength.

The transmittance attenuation factor $T_{\lambda }$ is the ratio of output to input intensity at wavelength *λ* [19,20]:

(2) $$T_{\lambda }=\frac{I}{I_{0}}=e^{-\mu_{\lambda }d}$$

Furthermore, the medium attenuation coefficient $\mu_{\lambda }$ can generally be stated based on Equation (2), where *I* and *I*_o_ are obtained from the measurement results and *d* is the thickness of the specimen medium.

(3) $$\mu_{\lambda }=\frac{-ln\frac{I}{I_{0}}}{d}$$

Then, Equation (3) can be rewritten as

(4) $$\mu_{\lambda }=\frac{ln{T_{\lambda }}^{-1}}{d}$$

In this research, the biological tissues are represented by chicken tissues. To begin the theoretical analysis, the chicken tissue specimen was arranged in stacks throughout the area. In general, the total thickness of the chicken specimen, which was the object of the study, was denoted as *d*. Assuming that the object undergoing testing contained three tissues, each tissue was distinguished by an index, where *m* represents meat, *b* represents bone, and *s* represents skin. Furthermore, the thickness of the chicken meat is symbolized as *d_m_*, that of the chicken bone as *d_b_*, and that of the chicken skin as *d_s_*. The total thickness of the object could, thus, be expressed as follows:

(5) $$d=d_{m}+d_{b}+d_{s}$$

The transmittance attenuation factor $T_{\lambda }$ of the specimen could generally be expressed as follows [21]:

(6) $$T_{\lambda }=e^{-\mu_{m\lambda }d_{m}}\cdot e^{-{\mu_{b\lambda }d}_{b}}\cdot e^{-\mu_{s\lambda }d_{s}}$$

where

$\mu_{m\lambda }$: chicken meat attenuation coefficient at wavelength λ;
$\mu_{b\lambda }$∶ chicken bone attenuation coefficient at wavelength λ;
$\mu_{s\lambda }$: chicken skin attenuation coefficient at wavelength λ;
$T_{\lambda }$ factor is obtained based on the $\frac{I}{I_{0}}$ measurement, as shown in Equation (2).

The $T_{\lambda }$ factor needed to be transformed into a linear equation. This was performed by a natural logarithmic transformation (*ln*) over Equation (6). Furthermore, by assuming that the thickness of each tissue varied across the specimen area, the linear natural logarithmic equation of transmittance attenuation factor $\;T_{\lambda }$ at every coordinate  (x,y) on the 2D image could be expressed as follows:

(7) $$\ln T_{\lambda }(x,y)=-\left[\mu_{m\lambda }d_{m}(x,y)+\mu_{b\lambda }d_{b}(x,y)+\mu_{s\lambda }d_{s}(x,y)\right]$$

Equation (7) represents a linear equation at each image coordinate point or pixel (x,y) taken at a certain monochromatic wavelength λ. In this experiment, the specimen object being analyzed had three different tissues. To derive the thickness of each tissue down to the pixel point, according to the rules of linear equation analysis, we required three similar transmittance image frames of the specimen object taken by three different λ monochromatic light sources. In this manner, the pixel coordinates (x,y) of each image frame were represented by three linear equations taken on three different wavelengths [22], as shown in the following three linear equations:

(8) $$\ln T_{\lambda 1}(x,y)=-\left[\mu_{m\lambda 1}d_{m}(x,y)+\mu_{b\lambda 1}d_{b}(x,y)+\mu_{s\lambda 1}d_{s}(x,y)\right]$$

(9) $$\ln T_{\lambda 2}(x,y)=-\left[\mu_{m\lambda 2}d_{m}(x,y)+\mu_{b\lambda 2}d_{b}(x,y)+\mu_{s\lambda 2}d_{s}(x,y)\right]$$

(10) $$\ln T_{\lambda 3}(x,y)=-\left[\mu_{m\lambda 3}d_{m}\left(x,y\right)+\mu_{b\lambda 3}d_{b}\left(x,y\right)+\mu_{s\lambda 3}d_{s}\left(x,y\right)\right]$$

where $\lambda_{1}$, $\lambda_{2}$, and $\lambda_{3}$ represent three different monochromatic wavelengths in the NIR spectrum. Equations (8)–(10) above can be arranged and rewritten as a linear matrix multiplication at pixel (x,y), as follows:

(11a) $$\left[\begin{array}{ccc} \mu_{m\lambda 1} & \mu_{b\lambda 1} & \mu_{s\lambda 1}\\ \mu_{m\lambda 2} & \mu_{b\lambda 2} & \mu_{s\lambda 2}\\ \mu_{m\lambda 3} & \mu_{b\lambda 3} & \mu_{s\lambda 3} \end{array}\right]\left[\begin{array}{c} d_{m}(x,y)\\ d_{b}(x,y)\\ d_{s}(x,y) \end{array}\right]=\left[\begin{array}{c} \ln T_{\lambda 1}^{-1}(x,y)\\ \ln T_{\lambda 2}^{-1}(x,y)\\ \ln T_{\lambda 3}^{-1}(x,y) \end{array}\right]$$

Furthermore, Equation (11a) can be simplified into the following form:

(11b) $$\left[\mu_{k\lambda }\right]\left[d_{k}(x,y)\right]=\left[\ln T_{\lambda }^{-1}(x,y)\right]$$

where $\left[\mu_{k\lambda }\right]$ refers to the tissue attenuation coefficient matrix for three types of chicken tissues at three different wavelengths, which can be obtained by measuring during the characterization steps. Furthermore, $\left[d_{k}(x,y)\right]$ refers to the tissue thickness vectors of the chicken tissue specimen at the pixel coordinates (x,y) of the images, whereas $\left[\ln T_{\lambda }^{-1}(x,y)\right]$ refers to the vector of the natural logarithm of inverse transmittance attenuation of the three images at the pixel coordinates (x,y).

**Figure 1:**
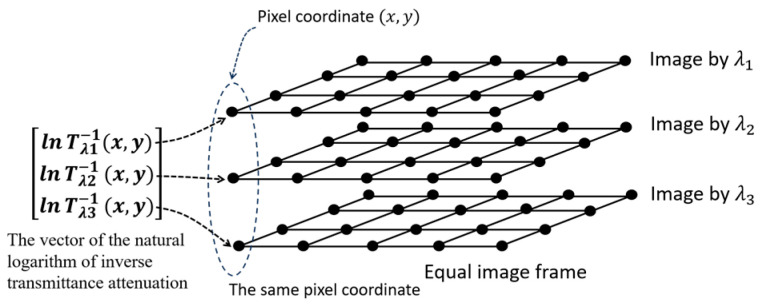
Illustration of how to construct the $\left[\ln T_{\lambda }^{-1}(x,y)\right]$ vector from three biological images taken with three different monochromatic light sources with equal frames.

The following Figure 1 illustrates the method of constructing the $\left[\ln T_{\lambda }^{-1}(x,y)\right]$ vector in Equation (11).

Furthermore, to obtain each tissue thickness value of the chicken specimen at coordinate pixels (x,y) of the object image, or simply the tissue thickness vector $\left[d_{k}(x,y)\right]$, Equation (11) must be inverted and arranged as follows:

(12a) $$\left[d_{k}(x,y)\right]={\left[\mu_{k\lambda }\right]}^{-1}\left[\ln T_{\lambda }^{-1}(x,y)\right]$$

or it can be rewritten in the following complete form:

(12b) $$\left[d_{k}\left(x,y\right)\right]=\left[\begin{array}{c} d_{m}(x,y)\\ d_{b}(x,y)\\ d_{s}(x,y) \end{array}\right]={\left[\begin{array}{ccc} \mu_{m\lambda 1} & \mu_{b\lambda 1} & \mu_{s\lambda 1}\\ \mu_{m\lambda 2} & \mu_{b\lambda 2} & \mu_{s\lambda 2}\\ \mu_{m\lambda 3} & \mu_{b\lambda 3} & \mu_{s\lambda 3} \end{array}\right]}^{-1}\left[\begin{array}{c} \ln T_{\lambda 1}^{-1}(x,y)\\ \ln T_{\lambda 2}^{-1}(x,y)\\ \ln T_{\lambda 3}^{-1}(x,y) \end{array}\right]$$

**Figure 2:**
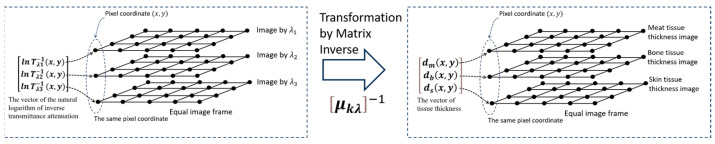
Transformation illustration from $\left[\ln T_{\lambda }^{-1}(x,y)\right]$ to $\left[d_{k}(x,y)\right]$ by using ${\left[\mu_{k\lambda }\right]}^{-1}$.

Figure 2, below, illustrates the transformation from $\left[\ln T_{\lambda }^{-1}(x,y)\right],$ the natural logarithm of the inverse transmittance attenuation vector, to $\left[d_{k}(x,y)\right]$, the tissue thickness vectors.

### 2.2. Attenuation Coefficient Characterizations

As written in Equation (12a,b), the tissue thicknesses at the point (x,y) are represented by the vector $\left[d_{k}(x,y)\right],$ where the index k represents the number of tissues in the specimen, which, in this research experiment, was k=3. To obtain $\left[d_{k}(x,y)\right]$, it is required that the tissue attenuation coefficient matrix inverse ${\left[\mu_{k\lambda }\right]}^{-1}$ be used to transform it from $\left[\ln T_{\lambda }^{-1}(x,y)\right]$. In constructing the $\left[\mu_{k\lambda }\right]$ matrix, first of all, we require the attenuation coefficient characterizations of each tissue available in the specimen, characterized at three different NIR wavelengths. The characterized tissue attenuation coefficients are then arranged to build the tissue attenuation coefficient matrix $\left[\mu_{k\lambda }\right]$ and then the inverse ${\left[\mu_{k\lambda }\right]}^{-1},$ as required by Equation (12a,b). Each type of tissue was prepared and placed in a very thin glass box for the characterization process. The homogenous tissue specimens had a known uniform thickness, “d”. Next, we prepared a planar wavefront monochromatic light source. It is recommended to use a strong NIR semiconductor laser and pass through a beam expander to obtain a Gaussian profile close to a planar wavefront.

### 2.3. Natural Logarithm of Inverse Transmittance Attenuation

The second step of the image decomposition process is to obtain the natural logarithm of the inverse transmittance attenuation vector $\left[\ln T_{\lambda }^{-1}(x,y)\right]$ of each pixel coordinate in the image, as mentioned in Equations (11a,b) and (12a,b). The measurement process is different from the tissue attenuation coefficient characterization method. The difference is that, in the characterization of $\left[\mu_{k\lambda }\right]$, every specimen was one type of homogeneous tissue, and the thickness d was known to be uniform. However, to obtain $T_{\lambda }$ values, the actual biological tissue specimen was a stack of three different tissues, and it had varying thicknesses throughout the specimen area. However, both the attenuation coefficient $\left[\mu_{\lambda }\right]$ characterization and $\left[\ln T_{\lambda }^{-1}(x,y)\right]$ vector measurement were carried out with equal proportions of the three different NIR wavelengths.

The transmittance attenuation factor *T* at a pixel (x,y) and wavelength λ can generally be written as follows:

(13) $$T_{\lambda }(x,y)=\frac{I_{\lambda }(x,y)}{I_{0@\lambda }(x,y)}$$

where $I_{\lambda }(x,y)$ is the object transmittance image intensity at pixel (x,y) and $I_{0@\lambda }(x,y)$ is light source intensity at pixel (x,y); both were taken at a wavelength λ. The natural logarithm of the inverse transmittance attenuation vector of the three images taken at the same frame and by three different wavelengths at the coordinate (x,y) can be arranged and written as follows:

(14) $$\left[\ln T_{\lambda }^{-1}(x,y)\right]=\left[\begin{array}{c} \ln T_{\lambda 1}^{-1}(x,y)\\ \ln T_{\lambda 2}^{-1}(x,y)\\ \ln T_{\lambda 3}^{-1}(x,y) \end{array}\right]$$

Equations (13) and (14) describe a similar phenomenon to Equations (8)–(11) regarding the vector of the natural logarithm of inverse transmittance attenuation; however, there is a context difference: Equations (8)–(11) show the theoretical concept utilized to obtain the $\left[\ln T_{\lambda }^{-1}(x,y)\right]$ vector, while Equations (13) and (14) emphasize obtaining the vector from measurements.

### 2.4. Tissue Thickness Vector $\left[d_{k}(x,y)\right]$

The third step is obtaining the tissue thickness vector $\left[d_{k}(x,y)\right]$, as mentioned in Equation (12b), whose components are the tissue object’s thicknesses at the (x, y) image pixel coordinate. The tissue thickness vector has the following components: $d_{m}(x,y)$ is the thickness of the meat, $d_{b}(x,y)$ is the thickness of the bone, and $d_{s}(x,y)$ is the thickness of the skin at pixel (x,y).

### 2.5. Tissue Thickness Image Matrix $\left[D_{k}\right]$

The fourth or final step in the image decomposition process is compiling vector components with the same tissue index *k* and arranging them by coordinate position (x, y) to form a tissue thickness image matrix $\left[D_{k}\right],$ where k is an index that represents a tissue layer of either meat (m), bone (b), or skin (s). Each 2D tissue thickness image describes the thickness distribution of each tissue layer. The value at a given (x, y) coordinate represents the tissue thickness at the coordinate, as shown in the following 2D matrix definition:

(15) $$\left[D_{k}\right]=\left[\begin{array}{ccc} d_{k}\left(0,0\right) & \cdots & d_{k}\left(0,N-1\right)\\ \vdots & \vdots & \vdots \\ d_{k}\left(M-1,0\right) & \cdots & x_{k}\left(M-1,N-1\right) \end{array}\right]$$

where $\left[D_{k}\right]$ is a matrix with dimensions of the image frame, i.e., M×N pixels, which is equal to the original image size. Matrix $\left[D_{k}\right]$ represents the thickness image of the $k^{th}$ chicken tissue.

Matrix $\left[D_{k}\right]$ was the final goal of this transmittance image decomposition research, i.e., the thickness image matrix of each of k-many tissues contained in the image object. The thickness image matrices were processed from the k-many initial transmittance images on the same object, which were taken on the same frame using k-many different NIR wavelengths.

## 3. Experimental Setup

**Figure 3:**
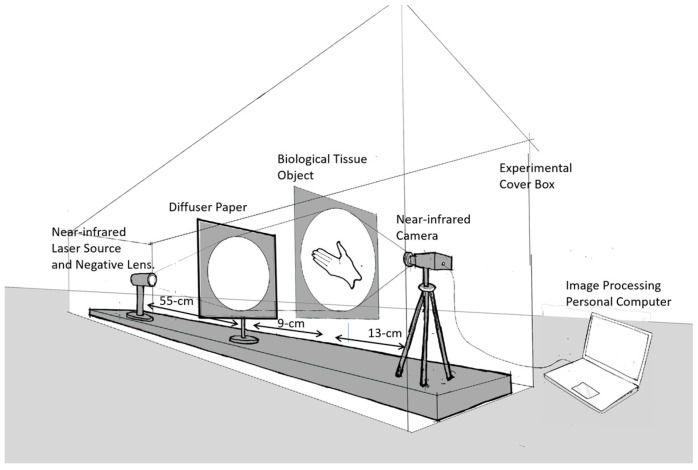
Experimental setup: the image is captured in transmittance mode.

In this research, the image was captured using a transmittance mode, where the light source was in the direction opposite to the camera and the object was in between, as shown in Figure 3 below.

The advantage of the transmittance capture method in the image capture process is that it allows us to obtain an overall profile of the biological organ tissues in the object. The main apparatus used was an infrared laser diode source with three different monochrome wavelengths: 780 nm (120 mW), 808 nm (300 mW), and 980 nm (100 mW). For the expanding beam diameter, a single negative lens with a focal length of 5 cm was used, followed by a thin light diffuser to suppress spatial speckle patterns on the light beam. We used an infrared-capable Thorlabs CMOS CS505MU camera equipped with an MVL5M23 lens to capture the image. For image acquisition, we developed an image processing program based on MATLAB (R2023a Update 3 (9.14.0.2286388)).

## 4. Results and Discussion

### 4.1. Tissue Attenuation Coefficient Characterizations and Constructing the Tissue Attenuation Coefficient Matrix $\left[\mu_{k\lambda }\right]$

**Figure 4:**
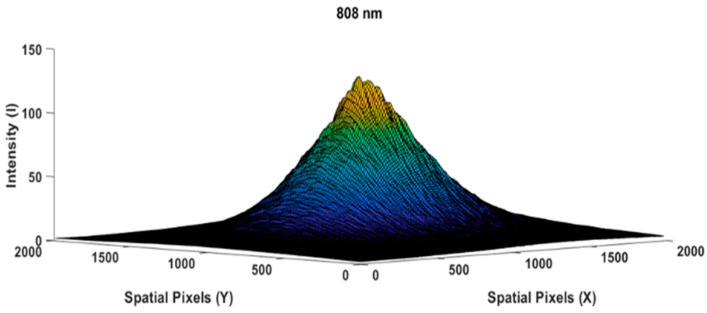
Reference beam profile image of an empty glass box, $I_{0@808}(x,y)$

**Figure 5:**
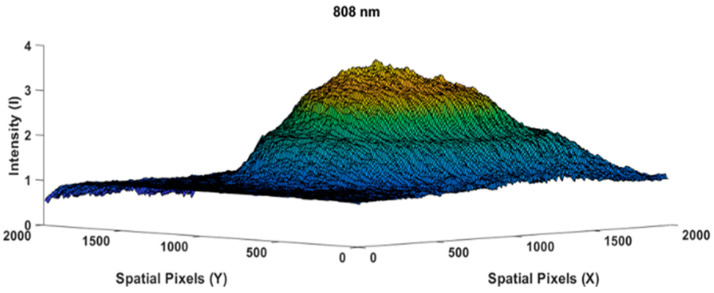
Beam profile image of the 0.5 cm thick chicken meat specimen $I_{cm0.5@808}\left(x,y\right),$ taken at an 808 nm wavelength.

The following Figure 4 is the result of measuring the light profile of the 808 nm wavelength with an empty glass case object used by the camera as a reference image. The beam profile appears very similar to a perfect Gaussian shape [23,24,25]; spatial speckling on the light beam was suppressed by applying a diffuser paper. Let us assume that the intensity distribution in Figure 4 is $I_{0@808}(x,y)$ as the reference image. While Figure 5 shows the measurement of the chicken meat object 0.5 cm in thickness, we used the same light intensity at a wavelength of 808 nm when measuring the reference beam profile image of Figure 4.

By referring to Equation (2), the transparency attenuation factor $T_{\lambda }$ at a point (x,y) from the chicken meat specimen measurement above could be obtained by dividing the Figure 5 image by the Figure 4 image; hence, Equation (2) can be rewritten as follows:

(16) $$T_{cm0.5@808}(x,y)=\frac{I_{cm0.5@808}(x,y)}{I_{0@808}(x,y)}$$

Furthermore, the attenuation coefficient of the chicken meat substance $\mu_{\lambda }$, as defined in Equation (4), can be rewritten as

(17) $$\mu_{cm@808}(x,y)=\frac{-ln(T_{cm0.5@808}(x,y))}{0.5cm}$$

**Figure 6:**
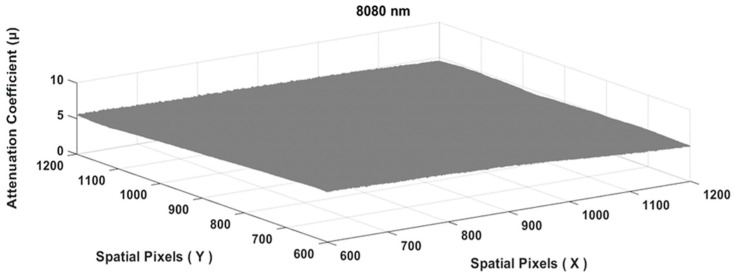
The tissue attenuation coefficient plane of the chicken meat specimen $\mu_{cm@808}(x,y)$ matrix.

The results of Equation (17) produced an image similar to a flat plane, as shown in Figure 6 below.

To obtain the final value of the attenuation coefficient at a particular wavelength, the entire matrix area of Figure 6 above was used to obtain a single value of $\bar{\mu_{cm@808}}$, as follows:

(18) $$\bar{\mu_{cm@808}}=\frac{\sum_{x,y}\mu_{cm@808}(x,y)}{\sum_{x,y}\left(1\right)}$$

**Figure 7:**
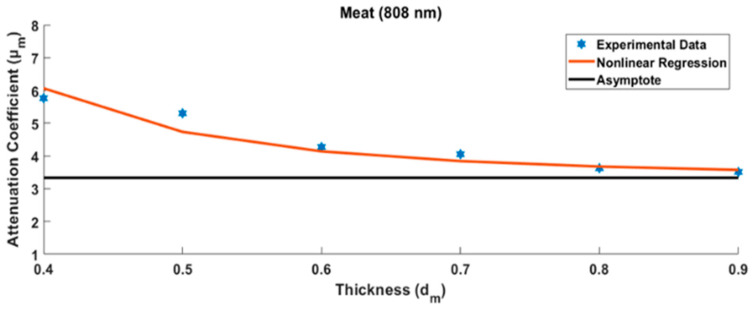
Several measurements were made to determine the final value of the chicken meat attenuation coefficient $\mu_{cm@808}(x,y)$ with various specimen thicknesses.

The chicken meat attenuation coefficient at the wavelength of 808 nm and specimen thickness of 0.5 cm (symbolized as $\mu_{cm@808})$ was 5.394 cm^−1^. In order to be able to obtain a reliable value, the measurements were conducted using different specimen thicknesses for the chicken meat, i.e., 0.5, 0.6, 0.7, 0.8, and 0.9 cm, as shown in Figure 7 below.

In principle, the measurement of the tissue specimen is influenced by the glass case that covers it. The thicker the measured tissue, the smaller the effect of the calculation error due to the glass case. Measurement data from several tissue specimens that grew thicker show asymptotic values. This asymptote value can be modeled, as will be discussed in the Discussion section. The same tissue of varying specimen thicknesses was further measured using two other different wavelengths. The other two tissue types (chicken skin and bone) were measured in the same way. Finally, nine attenuation coefficient values of three chicken tissues were arranged into a 3 × 3 $\left[\mu_{k\lambda }\right]$ matrix as shown in Table 1 below.

### 4.2. The Biological Tissue Image Decomposition Process

**Figure 8:**
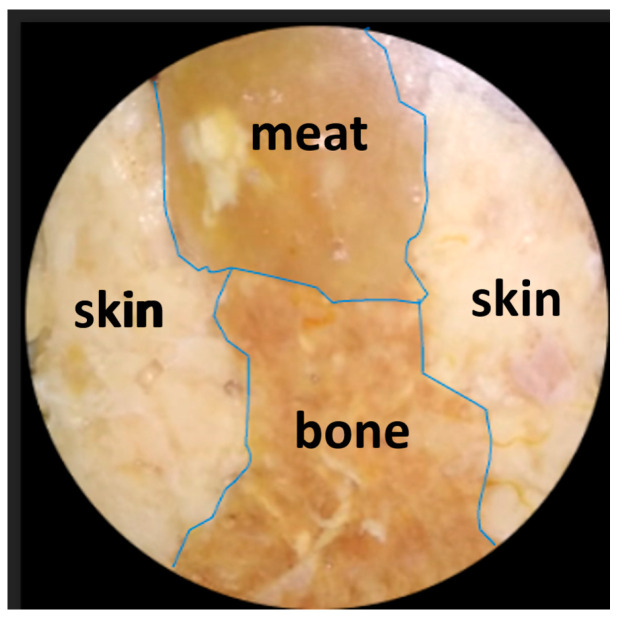
Image of the chicken tissue specimen consisting of three tissue substances, i.e., bone, meat, and skin, arranged side by side. The image was captured using a visible, polychromatic light source.

In order to prove the biological tissue image decomposition concept, the following analysis steps are carried out on a chicken tissue object consisting of three kinds of tissue—bone, meat, and skin—arranged side by side. First of all, the chicken tissue specimen was photographed with a visible polychromatic light source, as shown in Figure 8 below:

The same specimen was then photographed three times in the same frame using three different near-infrared monochromatic light sources. Each of the three images was further divided according to the reference image $I_{0@\lambda }(x,y)$ of each corresponding light source λ at the pixel level $\left(x,y\right),$ similarly to Equation (16). At each pixel (x,y), the three values were combined in a stack and formed into vectors $\left[\ln T_{\lambda }^{-1}(x,y)\right],$ as shown in Equation (14).

**Figure 9:**
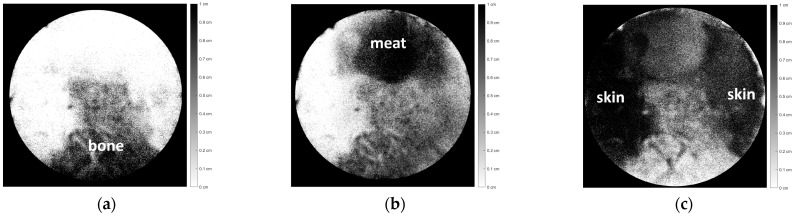
Image decomposition of Figure 8, consisting of three chicken tissues, i.e., (**a**) bone, (**b**) meat, and (**c**) skin.

Since the tissue attenuation coefficient matrix $\left[\mu_{k\lambda }\right]$ and the vector of the natural logarithm of inverse transmittance attenuation $\left[\ln T_{\lambda }^{-1}(x,y)\right]$ for each pixel in the entire image area had been obtained, the tissue thickness vectors $\left[d_{k}\left(x,y\right)\right]$ in the entire image area could then be obtained. The final results of the object image decomposition process were obtained as three images represented by a tissue thickness image matrix $\left[D_{k}\right]$, which corresponded to each tissue of the chicken specimen, as shown in Figure 9, as follows:

In this analysis, the tissue thickness is represented by a dark color. The thicker the tissue, the darker the color. If the tissue is thin, that area will be gray or even white if it has no thickness. Figure 9 (a) shows the bone section, (b) shows the meat section, and (c) shows the skin section. Figure 9a–c depicts each part of the tissue with relatively good accuracy with respect to Figure 8.

**Figure 10:**
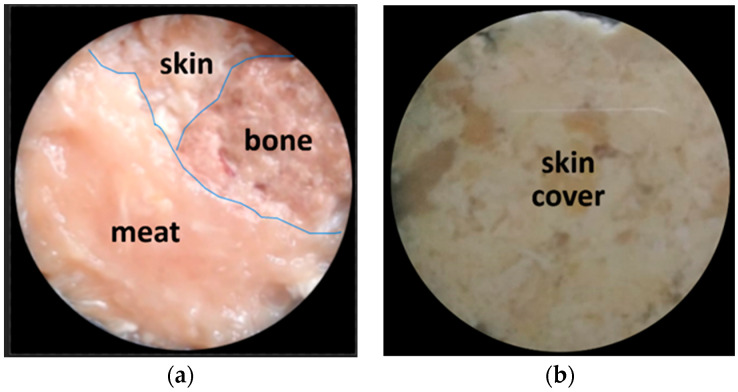
(**a**) A chicken tissue specimen consisting of three tissues, namely, bone, meat, and skin, arranged side by side. (**b**) Specimen is completely covered by chicken skin tissue. Images were captured using a visible polychromatic light source.

To further demonstrate the high potential of the proposed technique to decompose biological tissue object images, further experiments were performed with different biological object structures compared to Figure 8. As shown in Figure 10a, the chicken tissue specimen was composed of three substances, repeating the previous experiment. However, the specimen shown in Figure 10a was then covered completely by chicken skin tissue, as shown in Figure 10b below.

**Figure 11:**
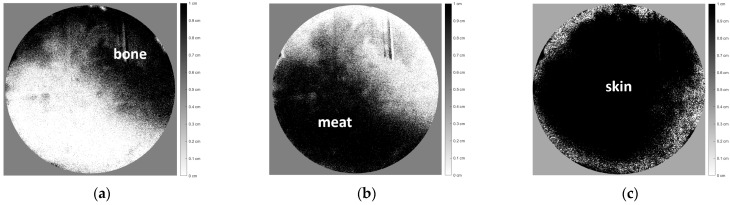
Image decomposition of Figure 10 according to the distribution thickness images of each chicken tissue, i.e., (**a**) bone, (**b**) meat, and (**c**) skin.

The images shown in Figure 10b were then decomposed into three images corresponding to each tissue of the chicken specimen, as shown in Figure 11 below.

Figure 11a–c shows the distribution thickness images of each chicken tissue, with relatively good accuracy corresponding to Figure 10, even though the object was completely covered by chicken skin tissue.

**Figure 12:**
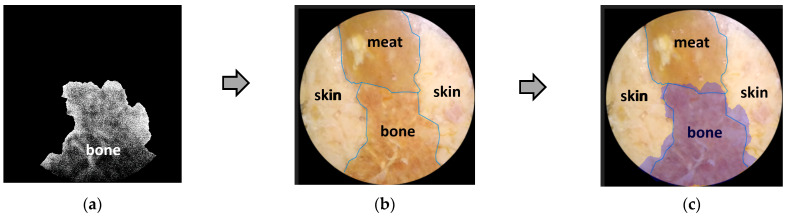
The illustration of procedural steps in estimating the quantitative accuracy of a decomposition image based on matching area. (**a**) Marking the edge boundary of bone tissue in Figure 9a; (**b**) overlaid on the bone area of Figure 8 and results in (**c**).

To further analyze the accuracy of this decomposition technique, besides a qualitative visual-based assessment, a quantitative assessment was also carried out by marking the edge boundaries of each tissue in Figure 8 and Figure 9a–c, as well as Figure 10 and Figure 11a–c. The tissue area image’s edge boundary is marked using the Image-Segmenter ToolBox in MATLAB software (R2023a Update 3). For instance, the detected bone tissue area, as shown in Figure 9a, was overlayed on top of the area in Figure 8 that corresponded to the bone tissue. The ratio of the overlapping area between the two to the area of the bone tissue section in Figure 8 represents the accuracy level of this decomposition process for bone tissue. The method for determining decomposition accuracy was limited to area ratios based on the Sorensen–Dice similarity criterion. The procedural steps carried out are illustrated in Figure 12 below:

Then, in the same way, a quantitative analysis was carried out for the other two tissues: skin and meat. The results of the quantitative analysis of Figure 8, Figure 9, Figure 10 and Figure 11 are summarized in the following Table 2.

Quantitatively, the matching area ratio value was above 60%, indicating that the new image decomposition technique is relatively reliable. This proves that the proposed method is promising for further development.

## 5. Discussion

As mentioned above, the main aim of this research was to decompose the transmittance image of a biological tissue object into several tissue thickness distribution images of each constituent tissue. The biological tissue object comprised three types of chicken tissue: meat, bone, and skin. The proposed image decomposition technique works based on the concept of transmission attenuation. In order to verify the newly proposed technique, there are at least three requirements that must be fulfilled. The first step is characterizing the transmission attenuation coefficient of each tissue making up the biological object with three different monochromatic NIR wavelengths. The second requirement is that the monochromatic NIR light sources must have a property in the form of a planar wavefront with minimum spatial noise. The third is that, in capturing the image, the camera must not be in a saturation state.

In the first step, i.e., characterization of the tissue attenuation coefficient $\left[\mu_{k\lambda }\right]$, the specimen was prepared by placing chicken tissues into a thin glass case with a thickness of approximately 0.05 cm. However, the glass case for packaging specimens reflected the light source, reducing light penetration. It is also possible to have multiple reflections on the glass packaging for thin specimens. This light reflectance was very influential, especially when characterizing absorption in very thin tissues. However, as the tissue thickened, the tissue absorption grew more dominant than the glass case reflection. Hence, the absorption measurement results became more stable, approaching the asymptotic value. Several measurements were made with different specimen thicknesses: 0.4 cm, 0.5 cm, 0.6 cm, 0.7 cm, 0.8 cm, and 0.9 cm. The thicker the specimen, the more it reduced the effect of the glass case [23], as shown in the measurement results in Figure 7 above.

In order to estimate the actual value of the attenuation coefficient, estimation models are needed based on the measurement results of several different specimen thicknesses, as shown in Figure 7 above. In this case, the estimation model was in the form of a nonlinear regression equation, as follows [26,27]:

(19) $$\mu_{k\lambda }=\beta_{0}+\beta_{1}\frac{1}{d_{k}^{3}}$$

where $d_{k}$ denotes the various thicknesses of the specimen, index k represents the kth type of biological tissue, $\beta_{0}$ is the estimated value of the asymptote, and $\beta_{1}$ is the coefficient for the inverse thickness of the specimen term to the power of three. The attenuation coefficient will be asymptotic to a certain value when the specimen is thicker. Therefore, the glass case effect can be ignored. Besides chicken meat, two other substances were characterized, i.e., chicken bone and skin, each with various specimen thicknesses, using three different monochromatic wavelengths: 780 nm, 808 nm, and 980 nm. Using the same method as that described above, the tissue attenuation coefficient was obtained for each type of chicken tissue at three different wavelengths. Finally, the tissue attenuation coefficient matrix ${\left[\mu_{k\lambda }\right]}_{3\times 3}$ was formed, as shown in Table 1.

Figure 9a–c, similar to the decomposition images of Figure 8, shows a slight inaccuracy. The boundary lines between tissues, as shown in Figure 9a–c, do not appear to be accurate, as shown in Figure 8. The image decomposition inaccuracy is suspected to have been caused by the determinant value of the tissue attenuation coefficient matrix $\left[\mu_{k\lambda }\right]$, $\det\left[\mu_{k\lambda }\right]=0.0887$, which was too low. The low determinant value indicates that the vector components in the matrix may have been very close to a linearly dependent condition [28,29]. A low determinant value can cause an inaccurate matrix inverse, which results in an inaccurate image decomposition process. This small determinant value may be caused by the three monochromatic wavelengths being too close to each other. This research used three monochromatic light sources, i.e., 780, 808, and 980 nm. Various variations in these three illumination NIR wavelengths will be carried out in future experiments to optimize the image decomposition process. It is essential to select the spectral region and wavelength variations that will be used to obtain much lower optical attenuation coefficient properties, with a high contrast ratio of attenuation coefficient between tissues and the wavelengths used. Exploring the far-infrared and terahertz spectral regions is also recommended. It is promising for this method to work effectively and have an optimal decomposition result.

As written in Equation (12a,b), some conditions are necessary in order to obtain the tissue thickness vector $\left[d_{k}(x,y)\right]$. The first required condition was that the tissue attenuation coefficient matrix $\left[\mu_{k\lambda }\right]$ needed to be an invertible square matrix. As is well known in linear algebra discussions, three linear equations are needed to solve three unknown variables, which, in this context, were three tissue thicknesses at the pixel coordinates $\left(x,y\right)$, where each of these linear equations was required to involve the sum of the multiplications between the tissue attenuation coefficients and the thickness of the corresponding layers, as formulated in Equations (8)–(10).

The second condition, as a consequence of the invertible matrix and pointed out by Equations (11) and (12a,b), states that if the biological chicken tissue specimen consists of three different tissues, then the tissue attenuation coefficient matrix $\left[\mu_{k\lambda }\right]$ must be in the form of a 3×3 square matrix. As shown in Equation (11), the $\left[\mu_{k\lambda }\right]$ matrix was composed of three rows. Each row consisted of three attenuation coefficients corresponding to three biological chicken tissues measured at the same wavelength. Different rows were measured at different monochromatic wavelengths.

The $\left[\mu_{k\lambda }\right]$ matrix, from another perspective, can also be expressed as a composition of three columns. Each column consists of three attenuation coefficients corresponding to the same biological tissue, measured at three different monochromatic wavelengths. Different columns correspond to the other tissues and were measured at three different monochromatic wavelengths. Equation (20) below illustrates what we have described above.

(20) $$\begin{array}{c} {\left[\mu_{\lambda }\right]}_{3\times 3}=\left[\begin{array}{ccc} \mu_{m\lambda 1} & \mu_{b\lambda 1} & \mu_{s\lambda 1}\\ \mu_{m\lambda 2} & \mu_{b\lambda 2} & \mu_{s\lambda 2}\\ \mu_{m\lambda 3} & \mu_{b\lambda 3} & \mu_{s\lambda 3} \end{array}\right]\;\;\;\;\;\begin{array}{c} \left[R_{1}\right]\\ \left[R_{2}\right]\\ \left[R_{3}\right] \end{array}\\ \begin{array}{ccc} \left[C_{1}\right] & \;\;\left[C_{2}\right] & \;\left[C_{3}\right] \end{array} \end{array}$$

As illustrated in Equation (20) above, the tissue attenuation coefficient matrix ${\left[\mu_{k\lambda }\right]}_{3\times 3}$ consisted of three-row vectors ${[R}_{1}]$, $\left[R_{2}\right]$, and ${[R}_{3}]$ or three-column vectors [$C_{1}]$, [$C_{2}]$, and [$C_{3}]$. The row vectors [$R_{1}]$, [$R_{2}]$, and [${CR}_{3}]$ were required to have a linearly independent relationship to qualify as an invertible matrix [28,29]. Likewise, column vectors C1, C2, and C3 needed to have the same relationship.

**Figure 13:**
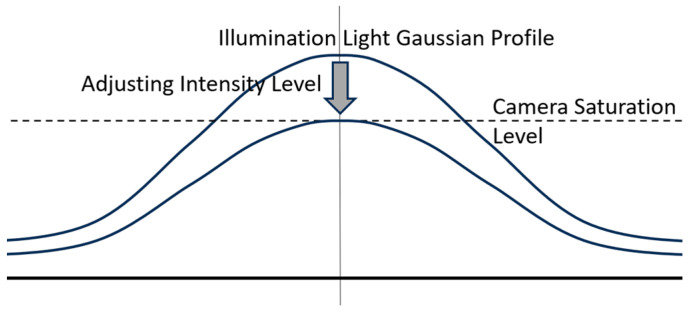
Illustration of setting the illumination intensity of the laser light source to not exceed the camera saturation threshold by adjusting the bias current in the laser semiconductor.

The third mandatory condition requirement is that the illumination intensity must be below the camera’s saturation threshold. In general, the laser light source used has an intensity level well above the saturation level of the camera. The intensity of the laser light source is adjusted so that the peak of the Gaussian profile distribution does not exceed the camera saturation threshold. This setting is conducted by adjusting the bias current of the laser semiconductor used, as shown in Figure 13 below.

## 6. Conclusions

In this research, a new technique of NIR-based transmittance image decomposition was developed for biological tissue specimens based on a matrix inverse method. The image was captured in transmittance mode. The experiment results show that the technique successfully decomposed the biological object image based on its components, i.e., different types of biological tissues. Although there are still some inaccuracies, as this paper represents initial research, this transmittance image decomposition technique holds great promise for the future of image instrumentation in the medical field. The image decomposition process can be improved by increasing the accuracy of tissue attenuation characterization and the selection of optimal optical spectral regions and wavelength illumination variations. Exploring the far-infrared and terahertz spectral regions is also recommended. Moreover, image decomposition accuracy can be improved even more by making the illumination wavefront as planar as possible. In the future, machine learning technology is expected to be able to further assist in optimizing the inverse matrix transformation process. Furthermore, as the ultimate goal, when the images are taken from various angles around the object, a 3D perspective tissue image of the observed object can be produced. This is fascinating because it is possible to provide another alternative to the 3D medical image analysis modality in a low-energy manner.

## Figures and Tables

**Table 1:** The tissue attenuation coefficient matrix $\left[\mu_{k\lambda }\right]$ of chicken tissues.

Chicken Tissue	$\lambda_{1}$ = 780 nm	$\lambda_{2}=$ 808 nm	$\lambda_{3}=$ 980 nm
Meat	4.004 ± 0.286	3.335 ± 0.274	3.134 ± 0.282
Skin	2.522 ± 0.245	2.454 ± 0.249	1.935 ± 0.188
Bone	4.758 ± 0.405	4.359 ± 0.343	3.619 ± 0.315

**Table 2:** Quantitative analysis of decomposition accuracy based on matching area ratio.

Chicken Tissue	Figure 8 and Figure 9 Accuracy	Figure 10 and Figure 11 Accuracy
Meat	75%	78%
Skin	85%	80%
Bone	91%	65%

## Data Availability

Data are contained within the article.

## References

[1] Thomson J.J. (1896). The Röntgen Rays. Nature.

[2] Jaszczak R.J., Coleman R.E., Lim C.B. (1980). SPECT: Single Photon Emission Computed Tomography. IEEE Trans. Nucl. Sci..

[3] Dorbala S., Ananthasubramaniam K., Armstrong I.S., Chareonthaitawee P., DePuey E.G., Einstein A.J., Gropler R.J., Holly T.A., Mahmarian J.J., Park M.-A. (2018). Single Photon Emission Computed Tomography (SPECT) Myocardial Perfusion Imaging Guidelines: Instrumentation, Acquisition, Processing, and Interpretation. J. Nucl. Cardiol..

[4] Maisey M.N., Bailey D.L., Townsend D.W., Valk P.E., Maisey M.N. (2005). Positron Emission Tomography in Clinical Medicine. Positron Emission Tomography: Basic Sciences.

[5] Hashemi R.H., Bradley W.G., Lisanti C.J. (2012). MRI: The Basics.

[6] Currie G., Iqbal B., Wheat J., Davidson R., Kiat H. (2011). Single photon emission computed tomography (SPECT)/computed tomography (CT): An introduction. Radiographer.

[7] Bailey D.L. (1998). Transmission scanning in emission tomography. Eur. J. Nucl. Med..

[8] Hielscher A., Bluestone A., Abdoulaev G., Klose A.D., Lasker J., Stewart M., Netz U., Beuthan J. (2002). Near-Infrared Diffuse Optical Tomography. Dis. Markers.

[9] Nammas W., Ligthart J.M., Karanasos A., Witberg K.T., Regar E. (2013). Optical coherence tomography for evaluation of coronary stents in vivo. Expert Rev. Cardiovasc. Ther..

[10] Strangman G., Boas D.A., Sutton J.P. (2002). Non-invasive neuroimaging using near-infrared light. Biol. Psychiatry.

[11] Xu Y., Iftimia N., Jiang H., Key L.L., Bolster M.B. (2001). Imaging of in vitro and in vivo bones and joints with continuous-wave diffuse optical tomography. Opt. Express.

[12] Xu Y., Iftimia N., Jiang H., Key L.L., Bolster M.B. (2002). Three-dimensional diffuse optical tomography of bones and joints. J. Biomed. Opt..

[13] Merlo S., Bello V., Bodo E., Pizzurro S. (2019). A VCSEL-Based NIR transillumination system for morpho-functional imaging. Sensors.

[14] Maqsood S., Javed U. (2020). Multi-modal medical image fusion based on two-scale image decomposition and sparse representation. Biomed. Signal Process. Control.

[15] Schultz A., Heikenfeld J., Kang H., Cheng W. (2011). 1000:1 contrast ratio transmissive electrowetting displays. J. Disp. Technol..

[16] Cheong W.-F., Prahl S.A., Welch A.J. (1990). A review of the optical properties of biological tissues. IEEE J. Quantum Electron..

[17] Shi L., Sordillo L.A., Rodríguez-Contreras A., Alfano R. (2016). Transmission in near-infrared optical windows for deep brain imaging. J. Biophotonics.

[18] Zhang L., Wang Z., Zhou M. (2010). Determination of the optical coefficients of biological tissue by neural network. J. Mod. Opt..

[19] Li W., Batteux F., Araujo S., Delpouve N., Saiter J.M., Tan L., Negahban M. (2016). Measurement of Beer-Lambert Attenuation Coefficient and Curing Kinetics Power Order: A Method Based on Rapid-Scan FTIR During Laser Curing on an ATR. Macromol. Symp..

[20] Mayerhöfer T.G., Pahlow S., Popp J. (2020). The Bouguer-Beer-Lambert law: Shining light on the obscure. ChemPhysChem.

[21] Liu B., Zhao J., Liu L. (2021). Applicability of Beer’s law in particulate system from random to regular arrangement: A numerical evaluation. J. Quant. Spectrosc. Radiat. Transf..

[22] Paul I., Mani A. (2018). A filtering strategy for the convergence of the Beer-Bouguer law for radiation extinction in random particulate media. Annu. Res. Breifs.

[23] Aminoto T., Priambodo P.S., Sudibyo H. (2022). Image Decomposition Technique Based on Near-Infrared Transmission. J. Imaging.

[24] Golovynskyi S., Golovynska I., Stepanova L.I., Datsenko O.I., Liu L., Qu J., Ohulchanskyy T.Y. (2018). Optical windows for head tissues in near-infrared and short-wave infrared regions: Approaching transcranial light applications. J. Biophotonics.

[25] Dytso A., Bustin R., Poor H.V., Shamai S. (2018). Analytical properties of generalized Gaussian distributions. J. Stat. Distrib. Appl..

[26] Mahaboob B., Praveen J.P., Rao B.A., Haranadh Y., Narayana C., Prakash G.B. (2020). A study on multiple linear regression using matrix calculus. Adv. Math. Sci. J..

[27] Konishi S. (2014). Introduction to Multivariate Analysis: Linear and Nonlinear Modeling.

[28] Anton H., Rorres C. (2013). Elementary Linear Algebra: Applications Version.

[29] Szabo F. (2015). The Linear Algebra Survival Guide: Illustrated with Mathematica.

